# Attenuated effective connectivity of large-scale brain networks in children with autism spectrum disorders

**DOI:** 10.3389/fnins.2022.987248

**Published:** 2022-11-29

**Authors:** Lei Wei, Yao Zhang, Wensheng Zhai, Huaning Wang, Junchao Zhang, Haojie Jin, Jianfei Feng, Qin Qin, Hao Xu, Baojuan Li, Jian Liu

**Affiliations:** ^1^Network Center, Air Force Medical University, Xi’an, China; ^2^Military Medical Center, Xijing Hospital, Air Force Medical University, Xi’an, China; ^3^School of Biomedical Engineering, Air Force Medical University, Xi’an, China; ^4^Department of Psychiatry, Xijing Hospital, Air Force Medical University, Xi’an, China

**Keywords:** autism spectrum disorder, brain network, dynamic causal modelling, effective connectivity, parametric empirical bayesian

## Abstract

**Introduction:**

Understanding the neurological basis of autism spectrum disorder (ASD) is important for the diagnosis and treatment of this mental disorder. Emerging evidence has suggested aberrant functional connectivity of large-scale brain networks in individuals with ASD. However, whether the effective connectivity which measures the causal interactions of these networks is also impaired in these patients remains unclear.

**Objects:**

The main purpose of this study was to investigate the effective connectivity of large-scale brain networks in patients with ASD during resting state.

**Materials and methods:**

The subjects were 42 autistic children and 127 age-matched normal children from the ABIDE II dataset. We investigated effective connectivity of 7 large-scale brain networks including visual network (VN), default mode network (DMN), cerebellum, sensorimotor network (SMN), auditory network (AN), salience network (SN), frontoparietal network (FPN), with spectral dynamic causality model (spDCM). Parametric empirical Bayesian (PEB) was used to perform second-level group analysis and furnished group commonalities and differences in effective connectivity. Furthermore, we analyzed the correlation between the strength of effective connectivity and patients’ clinical characteristics.

**Results:**

For both groups, SMN acted like a hub network which demonstrated dense effective connectivity with other large-scale brain network. We also observed significant causal interactions within the “triple networks” system, including DMN, SN and FPN. Compared with healthy controls, children with ASD showed decreased effective connectivity among some large-scale brain networks. These brain networks included VN, DMN, cerebellum, SMN, and FPN. In addition, we also found significant negative correlation between the strength of the effective connectivity from right angular gyrus (ANG_R) of DMN to left precentral gyrus (PreCG_L) of SMN and ADOS-G or ADOS-2 module 4 stereotyped behaviors and restricted interest total (ADOS_G_STEREO_BEHAV) scores.

**Conclusion:**

Our research provides new evidence for the pathogenesis of children with ASD from the perspective of effective connections within and between large-scale brain networks. The attenuated effective connectivity of brain networks may be a clinical neurobiological feature of ASD. Changes in effective connectivity of brain network in children with ASD may provide useful information for the diagnosis and treatment of the disease.

## Introduction

Since its first clinical description in the 1940s, autism spectrum disorder (ASD) has gone from a rare childhood-onset disorder to a pervasive, lifelong developmental disorder. As a neurodevelopmental deficit, the disorder is clinically characterized by impairments in social interaction, communication disorders, restricted interests, stereotyped and repetitive behavioral patterns, and in most patients, developmental delay (Belmonte et al., 2004). A data statistics in 2012 in the United States shows that the incidence rate of ASD is very high, with an average of 1 in 68 children suffering from ASD, and the prevalence rate of boys is 4.5 times that of girls (Christensen et al., 2016). Children with ASD may need long-term or even lifelong care. Therefore, ASD treatment incurs higher medical and educational costs, which is a huge financial burden for most families and society. However, the underlying pathophysiological and neurological basis of ASD remains unclear, and effective diagnosis and treatment of ASD is elusive (Courchesne et al., 2011).

Researchers are attempting to understand the neurological basis of ASD by identifying biomarkers associated with core defects (Mohammad-Rezazadeh et al., 2016). One of the most commonly used methods is functional magnetic resonance imaging (fMRI). A growing number of imaging studies involving different groups of children, adolescents, and adults have shown that ASD is a disorder of abnormal brain connections. These abnormal connections exist not only in separate brain networks, such as visual network (VN), default mode network (DMN), auditory network (AN), salience network (SN), and frontoparietal network (FPN), but also between different brain networks (Bi et al., 2018; Chen et al., 2021). In studies concerning VN, survey such as that conducted by Lombardo et al. (2019) found reduced functional connectivity between DMN and VN in patients with ASD from 12 to 48 months of age, and eye-tracking procedures revealed low connectivity between DMN and occipito-temporal cortex (OTC). In contract, another study found increased connectivity of language areas with posterior cingulate cortex (PCC) and visual areas in adolescents with ASD (Gao et al., 2019). About DMN, One study by Lynch et al. (2013) found that children with ASD exhibited high connectivity of the posterior cingulate and retrosplenial cortices, yet their precuneus showed localized hypo-connectivity with the visual cortex, basal ganglia, and posteromedial cortex. Besides, in a study of patients with ASD from Autism Brain Imaging Data Exchange I (ABIDE I), it was found that connectivity of the precuneus, posterior cingulate gyrus and the medial prefrontal gyrus was decreased in DMN (Yao et al., 2016). Similarly, Funakoshi et al. (2016) found reduced functional connectivity in the bilateral inferior parietal lobule and posterior cingulate cortex of DMN in children with ASD. In studies of the cerebellum, one study found reduced gray matter in the inferior cerebellar vermis (lobule IX), left lobule VIIIB, and right Crux I in patients with ASD (Stoodley, 2014). In another study, functional connectivity between the right cerebellar region and the supratentorial regulatory language areas was absent in adolescents with ASD (Verly et al., 2014). Conversely, a new study demonstrated that adolescents with ASD had cerebro-cerebellar functional overconnectivity (Khan et al., 2015). In an analysis of AN (Linke et al., 2018), the interhemispheric connectivity of the left and right auditory combined ROI decreased, and the connectivity between auditory cortical areas and the thalamus increased in the adolescents with ASD. In a study investigating SN, Zenghui et al. (2019) reported that adolescents with ASD had higher functional connectivity between the right dorsolateral prefrontal cortex and the left superior frontal gyrus, the middle frontal gyrus and the anterior cingulate cortex. About FPN, it was found that patients with ASD had greater activation in the right middle frontal gyrus and anterior cingulate cortex and less activation in the bilateral middle frontal, left inferior frontal gyrus, right inferior parietal lobule, and precuneus (May and Kana, 2020). In addition, data from several studies suggest that connections between different brain networks were both increased and decreased in patients with ASD. Compared with the control group, the connectivity between VN and sensorimotor network (SMN) and between the posterior part of the cerebellum and the occipital and parietal cortices was enhanced, while the connectivity between VN and AN, between the lateral cerebellum and the pre-frontal and other relevant cortical regions was reduced (Wang et al., 2019; Chen et al., 2021). Another study found that adolescents with ASD had varying increases or decreases in functional connectivity in eight brain networks, including DMN, VN, AN and so on (Bi et al., 2018). In conclusion, these studies show that the connections between the brain and cerebellum, between VN and SMN, and those related to AN in autistic patients show higher functional connectivity compared with the control group (Khan et al., 2015; Linke et al., 2018; Chen et al., 2021). However, the connection between VN and AN presents lower functional connectivity (Wang et al., 2019). In addition, the functional connection between DMN and VN, as well as the functional connection related to FPN, both increases and decreases (Lynch et al., 2013; Gao et al., 2019; Lombardo et al., 2019; May and Kana, 2020). These abnormal connections may be related to the special clinical symptoms of patients with ASD, such as social interaction disorder, communication disorder, limited interest and repetitive behavior patterns.

Functional connectivity in the brain is defined as the correlation of spontaneous functional activity between different brain regions (Harikumar et al., 2021), and refers to the time-dependence of neural activity signals in spatially disconnected brain regions (Chiang et al., 2016). It has been widely used to assess the functional interactions between different brain regions. It has also been widely used by researchers in previous studies on ASD. However, functional connectivity cannot portray the directional information of connections between brain regions (Friston et al., 2003). Effective connectivity has been defined as the effect of neurological activity in one brain region on another brain region. Unlike functional connectivity, effective connectivity can estimate the direction of information transfer of neural activity (Rajabioun et al., 2020), which helps to understand the brain mechanisms behind neural activity. Dynamic causal modelling (DCM) (Friston et al., 2003) is a common approach to effective connectivity. Li et al. (2011) proposed a stochastic DCM model. By introducing noise as the input signal of the model, the effective connectivity parameters under noise interference were estimated, thus allowing the DCM to be used for resting-state fMRI analysis. To solve the problem of DCM model instability in the time domain inversion, Friston et al. (2014) proposed the spectral dynamic causal modelling (spDCM) method, which can effectively reduce the complexity of model estimation by transferring the model to the frequency domain for inversion through frequency domain approximation. In addition, the stability and accuracy of spDCM model estimation are improved compared with stochastic DCM (Razi et al., 2015). Subsequently, to address some problems in the DCM process, such as local maxima in Bayesian model comparisons, Friston et al. (2016) proposed a Bayesian model reduction from the classical random effects model to a parametric empirical Bayesian (PEB) model using only the full model. By using the posterior density of the full model, the reduced model can be inverted by Bayesian methods.

In this study, we investigated the effective connectivity between different regions of each brain network in children with ASD. We used the public dataset KKI, which is part of the Autism Brain Imaging Data Exchange II (ABIDE II), which was created with the support of the National Institute of Mental Health and provides better phenotypic characteristics (Di Martino et al., 2017). In addition, ABIDE II includes a series of psychiatric variables, which can help us understand the neural correlates of psychopathology. Through spDCM, we investigated the effective connectivity of 7 large-scale brain networks in 42 children with ASD and 127 age-matched healthy controls. First, the regions of different brain networks were identified by spatially independent component analysis (McKeown and Sejnowski, 1998). Then, the effective connectivity of the regions of 7 large-scale brain networks were characterized using spDCM and Bayesian model reduction to find the directional and causal relationships of neural activity between regions. In addition, we analyzed the correlation between the effective connectivity between regions and some clinical indicators in children.

## Materials and methods

### Participants

We used the KKI dataset in this study. The KKI dataset collected from 211 children aged 8 to 13 years, including 56 children with ASD and 155 children in the typical controls (TC) group. Participants were enlisted as part of an ongoing study at the Kennedy Krieger Institute’s Center for Neurodevelopmental and Imaging Research. Informed consents were provided by the children’s parents or guardians, and consents were provided by children. They had a Wechsler Intelligence Scale for Children-IV (WISC-IV) (Wechsler, 2003) or WISC-V (Wechsler, 2014) full scale IQ greater than 80. Children were excluded if they had a definite neurological disease, severe chronic illness, severe visual impairment, alcohol or drug dependence, conditions that prohibit or make it difficult to obtain MRI, and were at developmental level 3 or higher on the Physical Development Scale (Carskadon and Acebo, 1993). The diagnosis of ASD was confirmed using the Autism Diagnostic Interview-Revised (ADI-R) (Lord et al., 1994) or the Autism Diagnostic Observation Schedule-2nd edition (ADOS-2) (Lord et al., 2012) module 3. The WISC-IV or WISC-V were used to assess intelligence. The Edinburgh Handedness Inventory (Oldfield, 1971) was used to determine handiness.

### Data acquisition

Scans were performed at the F.M. Kirby Research Center for Functional Brain Imaging using one of two Philips 3T scanners with an 8- or 32-channel phased-array head coil. Resting-state fMRI scans were collected before or after the structural scans. During resting-state scans, children were asked to relax and focus on a crosshair while remaining as still as possible. The scanning parameters of Resting-state fMRI scans were: repetition time = 2,500 ms, echo time = 30 ms, field of view = 256 mm x 256 mm, matrix = 96 × 96, flip angle = 75°, number of slices = 47, slice thickness = 3 mm, inter-slice gap = 0 mm. The scanning parameters of structural scans were: repetition time = 8.0 ms, echo time = 3.7 ms, field of view = 256 mm × 256 mm, matrix = 256 × 256, flip angle = 8°, slice thickness = 1 mm.

### Preprocessing

**FIGURE 1:**
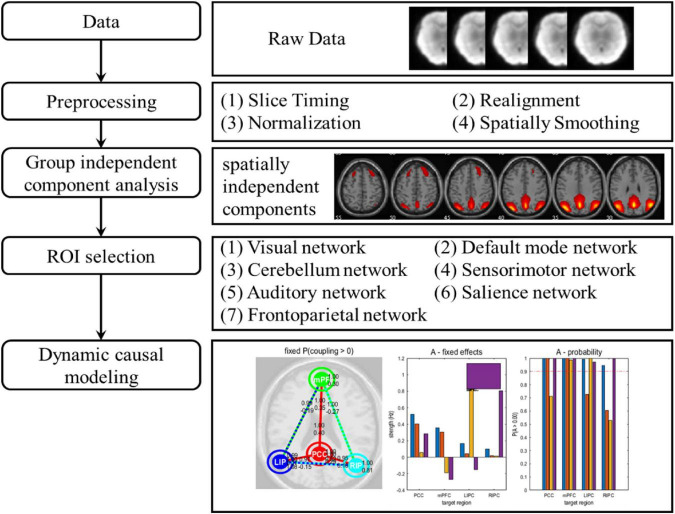
The data analysis pipeline. After data acquisition and preprocessing, the data were divided into 7 different brain networks through group independent component analysis. For each brain network, selected the corresponding regions of interest. Finally, dynamic causal modelling of all brain networks was carried out.

The preprocessing of data analysis is shown in Figure 1. Data processing was performed with the GRETNA toolbox (v2.0.0) (Wang et al., 2015) based on SPM12. In general, functional preprocessing included the following four steps. (1) Slice Timing: corrected the difference in acquisition time between slices in each volume; (2) Realignment: used rigid body transformation to correct for head motion; (3) Normalization: transformed brain of each subject into a standardized space defined by the Montreal Neurological Institute (MNI); (4) Spatially Smoothing: spatially smoothed data using a Gaussian filter with a full width at half maximum (FWHM) of 6 mm.

Some children were excluded from analysis after preprocessing due to poor normalization and excessive head motion. In addition, four children were excluded because the subsequent spDCM process did not converge. Therefore, a total of 14 children with ASD and 28 TC children were excluded, and the final sample included 42 children with ASD and 127 TC children. The demographic and clinical data of the subjects of each dataset are shown in Table 1.

**TABLE 1:** Demographic and clinical data of the subjects.

	ASD (*N* = 42)	TC (*N* = 127)	*p*-value
Sex (Male/Female)	29/13	75/52	0.2485
Age	10.48 ± 1.41	10.36 ± 1.14	0.5846
ADOS_G_TOTAL	13.72 ± 3.18	–	–
ADOS_G_COMM	3.44 ± 1.07	–	–
ADOS_G_SOCIAL	7.67 ± 2.19	–	–
ADOS_G_STEREO_BEHAV	2.61 ± 1.38	–	–

### Group independent component analysis

The preprocessed data were subjected to independent component analysis (ICA) using the Group ICA of fMRI Toolbox (GIFT) software package.^1^ The GIFT software first estimated and decomposed each subject’s fMRI image into 33 spatially independent components. Then performed back reconstruction on each subject to obtain the corresponding ICA map. According to the mean image of the spatially independent components of all the subjects, all the independent components are sorted by the ICA independent component template (Smith et al., 2009), and each brain network that matches the template was selected as the independent component of brain network. There are 7 selected brain networks, including VN, DMN, cerebellum, SMN, AN, SN, and FPN.

### Selection of regions of interest

Next, the corresponding regions of interest (ROI) in each of the identified brain networks were selected. Frist defined the ROI as a sphere with a radius of 6 mm, and used xjView^2^ to determine the coordinates of the center of each sphere. Then 24 ROIs corresponding to 7 brain networks were selected using the AAL standard brain template provided by the Montreal Neurological Institute (MNI). The peak value of each ROI was used as the coordinate of this ROI. The coordinates of different brain networks are shown in Table 2. The name of each ROI was represented by the corresponding abbreviation of the brain area in the AAL template.

**TABLE 2:** The coordinates of different brain networks.

Brain networks	ROI	Abbreviation	Coordinate
VN	Calcarine fissure and surrounding cortex	CAL_L	−6 −76 10
	Inferior occipital gyrus	IOG_R	28 −94 −4
	Middle occipital gyrus	MOG_L	−28 −94 −4
	Superior parietal gyrus	SPG_L	−20 −72 54
	Superior parietal gyrus	SPG_R	20 −74 54
DMN	Precuneus	PCUN_L	2 −76 40
	Inferior parietal, but supramarginal and angular gyri	IPL_L	−32 −72 42
	Angular gyrus	ANG_R	36 −70 42
	Superior frontal gyrus, medial	SFGmed_L	0 58 10
Cerebellum	Pons	Pons	0 −42 −42
SMN	Postcentral gyrus	PoCG_R	58 −6 28
	Precentral gyrus	PreCG_L	−56 −8 30
	Precuneus	PCUN_L	−2 −48 76
AN	Superior temporal gyrus	STG_R	62 −22 16
	Supramarginal gyrus	SMG_L	−60 −22 16
SN	Middle frontal gyrus	MFG_L	−30 56 16
	Middle frontal gyrus	MFG_R	32 56 16
	Anterior cingulate and paracingulate gyri	ACG_L	0 30 28
	Superior temporal gyrus	STG	54 14 -4
	Temporal pole: superior temporal gyrus	TPOsup_L	−50 14 −6
FPN	Middle frontal gyrus	MFG_R	44 18 50
	Inferior parietal, but supramarginal and angular gyri	IPL_R	44 -56 56
	Middle frontal gyrus	MFG_L	−44 22 46
	Superior parietal gyrus	SPG_L	−32 −74 52

### Dynamic causal modeling and statistical analysis

To investigate the effective connectivity in brain networks, we used spDCM embedded in the SPM12. For each subject, a complete connectivity model is created that allows bidirectional connections between any two ROIs. The model did not include any external input because our analysis was based on resting-state fMRI data. The full connectivity model’s parameters are then estimated using spDCM. More free parameters will be generated as more ROIs are used to build fully connected models. We employ Bayesian model reduction (Friston et al., 2016). The classical stochastic effect model was first transformed into a parameter empirical Bayesian PEB model, and then Bayesian inversion of the reduced model was performed using the complete model’s posterior density.

Furthermore, to investigate the correlations between the effective connection and clinical data, we measured Pearson’s correlation coefficient between the effective connectivity and clinical data such as ADOS generic or ADOS-2 module 4 total (ADOS_G_TOTAL), ADOS-G or ADOS-2 module 4 communication total (ADOS_G_COMM), ADOS-G or ADOS-2 module 4 reciprocal social interaction total (ADOS_G_SOCIAL), and ADOS-G or ADOS-2 module 4 stereotyped behaviors and restricted interest total (ADOS_G_STEREO_BEHAV).

## Results

### Selected brain networks

**FIGURE 2:**
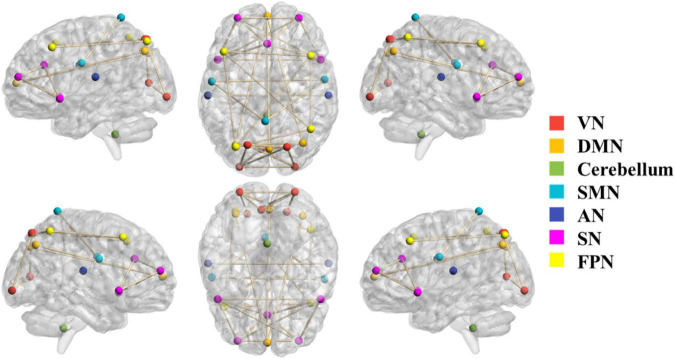
The selected 24 ROIs correspond to 7 brain networks. The color of the node represents the brain network to which the node belongs. The ROIs of each brain network are connected by lines.

The 24 ROIs corresponding to the 7 brain networks selected according to the ICA independent component template are shown in Figure 2. Different brain networks are marked with different colors to indicate their locations in the brain. The ROIs within each brain network are connected by lines. The 7 brain networks are evenly distributed in the brain, including the cerebellum.

### The common parts of the effective connectivity between the two groups

**FIGURE 3:**
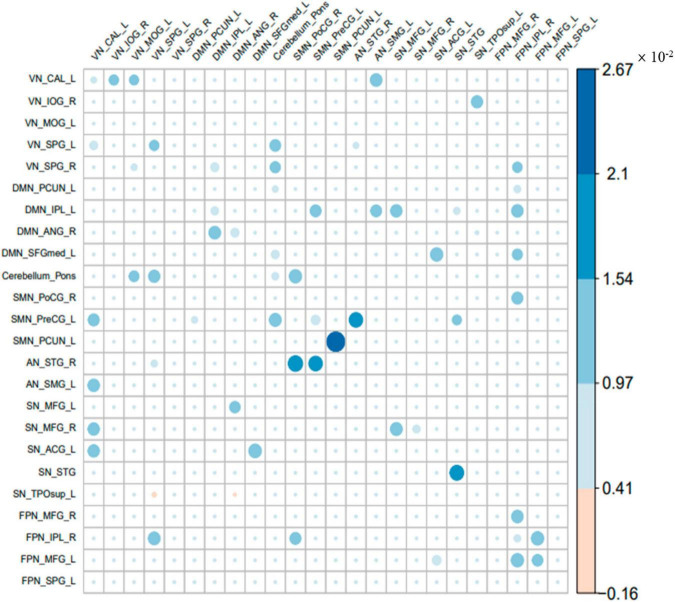
The common part of the effective connectivity between the two groups. The size of the circle indicates the size of the effective connectivity value.

The common parts of the effective connectivity between the two groups of 24 ROIs are shown in Figure 3. Each colored circle in the figure indicates that there is an effective connection between the brain regions of the corresponding row and column. The size of the colored circle corresponds to the size of the effective connectivity value, and the direction is from the brain region of the corresponding column to the brain region of the corresponding row. Blank indicates that there is no effective connection between the brain regions of the row and column. There was a total of 62 common effective connections between the 24 ROIs of the two groups. Among them, 60 effective connections were excitatory connections, and 2 connections were inhibitory connections, i.e., from VN_SPG_L to SN_TPOsup_L and from DMN_ANG_R to SN_TPOsup_L. The effective connectivity with the largest excitatory connection value was from SMN_PCUN_L to itself. It can be seen that the common effective connectivity is distributed in these 7 brain networks.

### The different parts of the effective connectivity between the two groups

**FIGURE 4:**
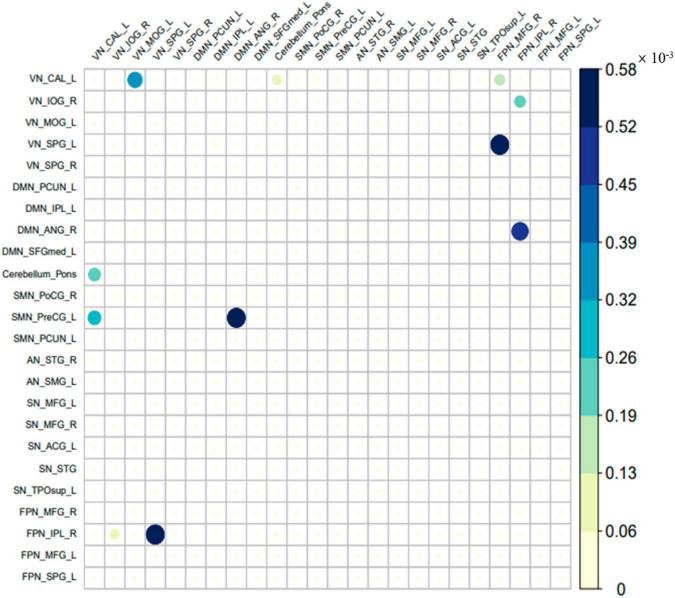
The different part of the effective connectivity between the two groups. The size of the circle indicates the size of the effective connectivity value.

The different parts of the effective connectivity between the two groups of 24 ROIs are shown in Figure 4. Consistent with Figure 3, each colored circle in the figure indicates that there is an effective connection between the brain regions of the corresponding rows and columns. The size of the colored circle corresponds to the size of the effective connectivity value, and the direction is from the brain region of the corresponding column to the brain region of the corresponding row. There were 11 different effective connections between the 24 ROIs of the two groups, and these 11 effective connections were all excitatory connections. The specific effective connectivity are shown in Table 3. It can be seen that the difference of effective connectivity was reflected in VN, cerebellum, DMN, SMN, and FPN, among which there were many excitatory connections between VN and FPN.

**TABLE 3:** The different parts of the effective connectivity between the two groups.

Number	Effective connectivity direction
1	VN_CAL_L - > Cerebellum_Pons
2	VN_CAL_L - > SMN_PreCG_L
3	VN_IOG_R - > FPN_IPL_R
4	VN_MOG_L - > VN_CAL_L
5	VN_SPG_L - > FPN_IPL_R
6	DMN_ANG_R - > SMN_PreCG_L
7	Cerebellum_Pons - > VN_CAL_L
8	FPN_MFG_R - > VN_CAL_L
9	FPN_MFG_R - > VN_SPG_L
10	FPN_IPL_R - > VN_IOG_R
11	FPN_IPL_R - > DMN_ANG_R

### Correlation

**FIGURE 5:**
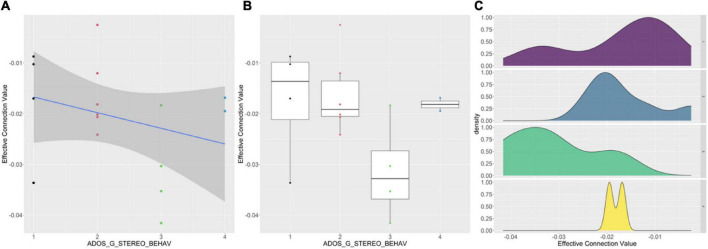
Correlations between effective connectivity values (from DMN_ANG_R to SMN_PreCG_L) and ADOS_G_STEREO_BEHAV. **(A)** The effective values and ADOS_G_STEREO_BEHAV has significant negative correlation. **(B)** The overall trend of ADOS_G_STEREO_BEHAV decreases as the effective connection value decreases. **(C)** For different ADOS_G_STEREO_BEHAV, the probability density distributions of the effective connection values are significantly different.

We analyzed the correlation between the clinical data of the patient group and the common parts of the effective connectivity between the two groups. And the correlation coefficient with significance level less than 0.05 was selected. A total of 17 effective connections was significantly correlated with clinical data. In addition, we analyzed the correlation between the clinical data of the patient group and the different parts of the effective connectivity between the two groups. And the correlation coefficient with significance level less than 0.05 was selected. We found a significant correlation between only one effective connection and one clinical data. We found an effective connectivity (from DMN_ANG_R to SMN_PreCG_L) and ADOS_G_STEREO_BEHAV had significant negative correlation. Figure 5 shows the details of relationships between the effective connectivity and ADOS_G_STEREO_BEHAV. From left to right are scatter plot, box plot and probability density plot separately.

## Discussion

In this study, we used spDCM method to compare the differences of effective connectivity between 24 ROIs corresponding to 7 large-scale brain networks between autistic children and matched healthy controls from the ABIDE II dataset. Three findings emerged from our analysis. First, for both groups, SMN is similar to a central network, which is closely and effectively connected to other brain networks. In addition, we also observed important causal interactions in the “triple network” system, including DMN, SN and FPN. Second, we found that children with ASD were characterized by attenuated effective connectivity among large-scale brain networks. Third, we observed significant correlation between the strength of the effective connectivity from ANG_R of DMN to PreCG_L of SMN and the ADOS_G_STEREO_BEHAV scores.

With the help of spDCM, we studied effective connectivity among large-scale brain networks in children with ASD. In both subject groups, we found that there were 62 significant effective connections in total, of which 60 were excitatory connections and 2 were inhibitory connections. Among these connections, the majority were related to the SMN. Our results are in line with previous studies of traditional functional connectivity (Du et al., 2021; Lepping et al., 2022). Furthermore, our studies extended previous finding by providing the directionality of the influences among these networks. In the connection within the DMN network, Yao et al. (2016) found that the connection between the precuneus, posterior cingulate gyrus and the medial prefrontal gyrus was reduced in DMN of children with ASD. Funakoshi et al. (2016) found that the functional connections of bilateral inferior parietal lobule and posterior cingulate cortex of DMN in children with ASD were reduced. We found that in DMN internal connections, effective connectivity involved IPL_L and ANG_R. They were from IPL_L to itself, from ANG_R to itself and from IPL_L to ANG_R, respectively. All three connections were excitatory connections. In the connection between different networks, Lombardo et al. (2019) found that the functional connection between DMN and VN in children with ASD was reduced. We further demonstrated in the current study, that the influences were mainly coming from the excitatory connection from IPL_L of DMN to SPG_R of VN. Chen et al. (2021) found hyperconnectivity between VN and SMN in children with ASD. We found that the effective connection between VN and SMN was from CAL_L of VN to PreCG_L of SMN.

In compared with healthy controls, we found attenuated effective connectivity in children with ASD. There were 11 effective connections, and these 11 effective connections were all excitatory connections. First, we found that the effective connection between the triple networks of ASD patients changed. This was consistent with previous studies that the dysfunction of one core network may affect the other two networks (Menon, 2011). Second, in the connection related to VN, we found that compared with normal children, the effective connectivity from MOG_L to CAL_L reduced in children with ASD. Keehn et al. (2021) found that the functional connection between the primary visual and extrastriate cortices within VM of ASD children increased. In addition, we found that the effective connectivity from CAL_L of VN to PreCG_L of SMN reduced in children with ASD. This was consistent with the results of Du et al. (2021). Their study showed that the functional connection between VN and SMN reduced in patients with ASD. And our results showed that part of VN had an excitatory effect on the part of SMN. Compared with normal children, children with ASD had 5 reduced effective connections between VN and FPN, namely, from IOG_R of VN to IPL_R of FPN, form SPG_L of VN to IPL_R of FPN, from MFG_R of FPN to CAL_L of VN, from MFG_R of FPN to SPG_L of VN, and from IPL_R of FPN to IOG_R of VN. This was consistent with previous studies. Yerys et al. (2019) found that there was a weak functional connection between FPN and the ventral attention subnetwork in patients with ASD. Third, in the connection related to DMN, we found that the connection between DMN and other brain networks was abnormal, in which the effective connectivity from ANG_R of DMN to PreCG_L of SMN reduced, and the effective connectivity from IPL_R of FPN to ANG_R of DMN reduced. This was consistent with previous functional connectivity studies. Previous studies have found underconnectivity and overconnectivity of DMN functional connections in children with ASD (Lynch et al., 2013; Yao et al., 2016).

In the study of the related mechanisms of brain regions, past studies have shown that visual impairment at birth or in early childhood can lead to psychological and emotional disorders (Wrzesinska et al., 2017). Our study further suggested that children with ASD may be associated with impaired vision. In connection with cerebellum, we found that the effective connectivity from CAL_L of VN to the pons of cerebellum reduced in children with ASD, and the effective connectivity of the opposite direction also reduced. These studies have shown that the effective connectivity between VN and cerebellum in children with ASD is abnormal. Anatomical and clinical studies have shown that the cerebellum is very important in motor learning and coordination and it supports cognitive, language and executive functions (Becker and Stoodley, 2013). Many clinical studies and animal model studies have shown that cerebellar dysfunction plays an important role in ASD (Tsai, 2016). So cerebellum may be related to the pathogenesis of ASD. Differences in DMN functional connectivity seem to be related to a wide range of social, communication, defects, and delays (Harikumar et al., 2021). The results of effective connection obtained in this paper prove this from another aspect. FPN was related to cognitive control processes, including behavioral response inhibition and working memory. And studies have proved that there are problems in VN of patients with ASD, so the abnormal connection between VN and FPN may be related to the pathogenesis of ASD.

In the study on effective connectivity of ASD, Rajabioun et al. (2020) found that in the resting state, the effective connectivity between active areas of children with ASD reduced, which was consistent with the results of reduced effective connectivity of children with ASD in this paper. In addition, we found that the effective connection from ANG_R of DMN to PreCG_L of SMN and ADOS_G_STEREO_BEHAV score had significant negative correlation. Rolls et al. (2020) found that the effective connectivity from the middle temporal gyrus to precuneus was negatively correlated with the total, communication and social scores of ASD. It can be speculated that the effective connectivity between brain regions of ASD patients is related to some clinical scores of ASD. The lower the effective connectivity, the higher the score of autistic children, and the more serious the ASD.

## Limitations

Our research has several limitations. First of all, we studied the effective connectivity in the resting state, we can study the effective connection in the task state and compare the differences in the future. Secondly, the age of the subjects we selected is between 8 and 13 years old, we can select subjects of other ages in the next study. Finally, the differences between patients before and after drug treatment should be considered in order to comprehensively understand the mechanism of ASD.

## Conclusion

In this study, different from the conventional functional connection, we use effective connectivity to study the relationship between different brain regions in children with ASD. The subjects were 42 autistic children and 127 age-matched normal children from the public data set KKI. The results showed that compared with normal children, the effective connectivity between different brain regions in ASD children were reduced. Our research provides new evidence for the pathogenesis of autistic children from the perspective of effective connections within and between large-scale brain networks. The decrease of effective connectivity of brain network may be a clinical neurobiological feature of ASD. The changes of effective connectivity of brain network in children with ASD may provide useful information for the diagnosis and treatment of the disease.

## Data availability statement

The original contributions presented in this study are included in the article/supplementary material, further inquiries can be directed to the corresponding authors.

## Ethics statement

The studies involving human participants were reviewed and approved by the Johns Hopkins Medical Institutional Review Board. Written informed consent to participate in this study was provided by the participants’ legal guardian/next of kin.

## Author contributions

BL, JL, and HX designed the study and provided important suggestions. LW, YZ, WZ, HW, JZ, HJ, JF, and QQ collected and collated the research data. LW, YZ, and WZ conducted data analysis and helped explain the results. LW, YZ, BL, and JL drafted the manuscript. All authors thoroughly reviewed and approved the manuscript for publication.

## Footnotes

1
https://trendscenter.org/software/gift/

2
http://www.alivelearn.net/xjview/
